# Adherence to National Dietary Guidelines in Association with Oral Health Impact on Quality of Life

**DOI:** 10.3390/nu10050527

**Published:** 2018-04-24

**Authors:** Valentina A. Andreeva, Emmanuelle Kesse-Guyot, Pilar Galan, Gilles Feron, Serge Hercberg, Martine Hennequin, Claire Sulmont-Rossé

**Affiliations:** 1 Equipe de Recherche en Epidémiologie Nutritionnelle (EREN), Centre de Recherche en Epidémiologie et Statistiques, Université Paris 13/INSERM U1153/INRA U1125/CNAM, COMUE Sorbonne Paris Cité F-93017 Bobigny, France; e.kesse@eren.smbh.univ-paris13.fr (E.K.-G.); p.galan@eren.smbh.univ-paris13.fr (P.G.); s.hercberg@eren.smbh.univ-paris13.fr (S.H.); 2 Centre des Sciences du Goût et de l’Alimentation, AgroSup Dijon, CNRS, INRA, Université Bourgogne Franche-Comté F-21000 Dijon, France; gilles.feron@inra.fr (G.F.); claire.sulmont-rosse@inra.fr (C.S.-R.); 3 Département de Santé Publique, Hôpital Avicenne, F-93017 Bobigny, France; 4 Université Clermont Auvergne, CROC EA 4847, F-63100 Clermont-Ferrand, France; martine.hennequin@uca.fr; 5 CHU de Clermont-Ferrand, Service d’Odontologie, F-63100 Clermont Ferrand, France

**Keywords:** diet quality, dietary guidelines, oral health, public health

## Abstract

We aimed to assess the association between oral health, in terms of its impact on quality of life, and diet quality expressed as adherence to dietary guidelines. We analyzed cross-sectional data from the French NutriNet-Santé general population-based e-cohort (N = 18,263 adults; mean age = 56.5 ± 13.8 years). The main independent variable, oral health-related quality of life, was assessed in 2016 with the GOHAI instrument (maximum score = 60). The main dependent variable, diet’s nutritional quality, was assessed with the mPNNS-GS score (maximum score = 13.5) measuring adherence to French dietary guidelines and computed on the basis of repeated 24-h dietary records. Multivariable linear regression models were fit. Mean GOHAI score was 54.5 ± 4.3 and mean mPNNS-GS score was 7.7 ± 1.6. Among participants aged 18–64 years, those scoring ≤50 on GOHAI (poor oral health with a detrimental impact on quality of life) were less likely to adhere to dietary guidelines than participants scoring 57–60 points (good oral health) (beta = −0.18, 95% CI: −0.26, −0.09; *p* < 0.0001). Among participants aged 65+ years, those scoring 51–56 points on GOHAI (average oral health with some negative impact on quality of life) were less likely to adhere to dietary guidelines than were participants scoring in the range 57–60 (beta = −0.23, 95% CI: −0.33, −0.13; *p* < 0.0001). The findings suggested modest age-dependent associations between oral health-related quality of life and diet quality. Confirmation is needed longitudinally with representative samples and accounting for diet quality evolution.

## 1. Introduction

Oral health implies being free from disorders, such as gum disease, sores, pain, and tooth decay/loss, that interfere with the capacity to speak and eat, and ultimately reduce general health status and quality of life (QOL) [1,2]. A strong correlation exists between oral health and general health, underscored by a host of common risk/protective factors (e.g., sugar intake, alcohol use, smoking, oral hygiene) [1,3]. Many general health conditions also have oral manifestations that increase the risk of oral disease [1]. For example, there is a well-established bidirectional association between periodontal disease, which is one of the most common chronic disorders, and diabetes mellitus [4]. Next, poor oral health has been associated with reduced dietary variety, inadequate intake of macro- and micronutrients, body mass index (BMI) outside the normal range, and risk of malnutrition [5,6,7,8]. Edentulous individuals and those with impaired dentition likely consume fewer vegetables and less fiber, while exhibiting increased intake of cholesterol, saturated fatty acids and calories, and reduced concentrations of serum beta carotene, folate, and vitamin C compared with their dentate counterparts [7,9,10]. Yet, methodological constraints of extant findings include the virtual absence of non-elderly individuals and paucity of research on overall diet quality. The few studies dealing with diet quality have mainly modeled the 1995 or 2005 Healthy Eating Index (HEI), measuring adherence to U.S. dietary guidelines [11,12]. Findings in a large sample of individuals aged ≥50 years revealed that those with impaired dentition had higher mean BMI and lower HEI (lower intake of vitamins A, B9, and C, decreased dietary variety, unfavorable cholesterol and sodium intake) compared with individuals with normal dentition [8]. In a sample of community-dwelling elderly aged ≥60 years, impaired dentition/major tooth loss was associated with lower HEI-2005, reflecting decreased consumption of fruit, vegetables, beans, and meat, and more energy from solid fat, alcohol and sugar, compared with those with a better dental status [13].

Nonetheless, up-to-date information on the link between oral health aspects and diet quality across age and research outside North America are lacking. Granted marked regional disparities, it has been estimated that 59% of Europeans do not have all their natural teeth and 15% have had difficulties over the past year in chewing, biting, or eating [14]. The objectives of this study were to assess the cross-sectional link between oral health aspects and diet quality. Specifically, we hypothesized a positive association between oral health-related QOL and adherence to national dietary guidelines. Oral health-related QOL was chosen as the exposure of interest owing to its pertinence to an epidemiological context and its discriminant potential as regards nutritional status [15]. The latter had been demonstrated by a study with men and women divided into three groups: those suffering from malnutrition, those at risk of malnutrition, and those with a normal nutritional status, as evaluated with the Mini-Nutritional Assessment [15,16].

## 2. Materials and Methods

### 2.1. NutriNet-Santé e-Cohort

This study is part of the multidisciplinary ALIMASSENS Project launched in France in 2014 [17]. The epidemiological component of the project is based on data from the ongoing NutriNet-Santé e-cohort launched in France in 2009 (www.etude-nutrinet-sante.fr) [18]. Volunteers aged ≥18 years with Internet access are recruited by means of traditional strategies (e.g., flyers) and multimedia campaigns. The NutriNet-Santé study was approved by the ethics committee of the French Institute for Health and Medical Research (No. 0000388FWA00005831) and by the National Commission on Informatics and Liberty (No. 908450 and No. 909216). Eligible participants provide informed consent and an electronic signature, and afterwards complete five baseline questionnaires: sociodemographics and lifestyle, health status, physical activity, anthropometrics, and diet. On a regular basis thereafter, self-report questionnaires on nutrition and/or health-related topics are sent. For this study, we selected participants residing in continental France who had completed the oral health questionnaire (described in Section 2.2) and at least three 24-h dietary records (described in Section 2.3).

### 2.2. Oral Health Assessment

An oral health questionnaire was administered between March and September 2016 to all active enrollees (*n* = 120,448). It included the General Oral Health Assessment Index (GOHAI) which is a self-report tool, initially designed to assess past-year oral health problems and oral health-related QOL of older adults, and later adapted for general use across age [19,20,21]. It consists of 12 items scored on a 5-point Likert scale (maximum score = 60) with lower scores corresponding to poorer oral health with a detrimental QOL impact. The following cutoffs have been established: score 57–60 as good oral health, score 51–56 as average oral health with some negative impact on QOL, and score ≤ 50 as poor oral health with a detrimental impact on QOL [19].The instrument also allows the assessment of three specific domains: physical function (four items, e.g., concerns about swallowing, speaking; maximum score = 20), psychosocial function (five items, e.g., concerns about self-image, avoiding social interactions; maximum score = 25), and discomfort/pain (three items; maximum score = 15). The French version of GOHAI has established validity and good psychometric properties (Cronbach’s alpha = 0.86, item-scale correlations range: 0.40–0.78) [20].

### 2.3. Diet Quality Assessment

The nutritional quality of diet was assessed in terms of adherence to French dietary guidelines established by the National Program on Nutrition and Health (PNNS) in 2001, with the most current update published in 2011 [22]. Individual dietary intake data were obtained from ≥3 24-h dietary records provided within 2.5 ears around the oral health questionnaire administration. Twice a year participants were asked to provide three 24-h records from non-consecutive days spread over a 2-week period. The user-friendly dietary record tool featured a food/beverage browser, a search engine, a user’s guide, and a built-in control system with cues and prompts designed to help minimize the chance of forgetting consumed items. For each food/beverage consumed over a period of 24 h, participants were asked to provide information about quantity/portion size, recipe/seasoning, and corresponding settings (time and place). Portion sizes were estimated using validated photographs [23]; a food composition table with >2000 different items was used to estimate macro- and micronutrient intake [24]. Mean intake values were calculated for each participant from all available 24-h dietary records over the selected period. Underreporting of dietary intake was identified using Black’s method [25] taking into account one’s sex, age, height, weight, physical activity, and basal metabolic rate, calculated via Schofield’s equations [26]. Individuals with extreme or likely energy underreporting were excluded from the analysis, as were those with <3 24-h records.

Adherence to PNNS dietary guidelines was measured by the *a priori* PNNS-Guideline Score (PNNS-GS). Its description and validation have been published [27,28]. The PNNS-GS is calculated on the basis of 13 components one of which pertains to physical activity and the remaining 12 to diet. The modified score (mPNNS-GS) is calculated on the basis of the 12 diet components only. Among those, 8 pertain to recommended intake quantities [fruit and vegetables (0–2 points); starchy foods (0–1 point); whole-grain foods (0–1 point); dairy (0–1 point); meat/poultry/eggs (0–1 point); seafood (0–1 point); vegetable fat (0–1 point); non-alcoholic beverages/water (0–1 point)]; four components pertain to recommended moderation in intake [added fat (0–1 point), salt (−0.5–1.5 points), sweets/desserts (−0.5–1 point), and alcohol (0–1 point)]. Details about categorization of the quantitative dietary data into the 12 mPNNS-GS components have been published [27]. Points are deducted if the reported energy intake >105% of estimated energy needs. The latter are based on an estimate of the basal metabolic rate calculated via Schofield’s equations [26]. Thus, mPNNS-GS is the sum of the 12 component scores minus the penalty points; maximum value = 13.5 points.

### 2.4. Covariates

We collected self-report sociodemographic (sex, age, marital status, education, occupation, income), lifestyle (physical activity, smoking), and health data (weight, height, diabetes, cancer, hypertension, cardiovascular diseases). Leisure-time physical activity was assessed with the International Physical Activity Questionnaire—Short Form, and scoring followed established protocol [29]. Missing data (education, *n* = 24; physical activity, *n* = 2; occupation, *n* = 260) were imputed using the mode of the respective variable. BMI was calculated as the weight (in kg) divided by the squared height (in m). Regarding health status, data on prevalent and incident events were collected. As enrollees were asked to provide this information on an annual basis for record updating purposes, we used information provided closest to the oral health questionnaire administration. Finally, we calculated and modeled as a covariate the interval between the date when demographic data were provided and the date when the oral health questionnaire was completed.

### 2.5. Statistical Analysis

Descriptive characteristics by the three-level GOHAI score are reported as percentages from chi-squared tests or mean (SD) from ANOVA, as appropriate. Overall GOHAI score and each of its three dimensions (physical, psychosocial, discomfort/pain) were the main independent variables; mPNNS-GS was the main dependent variable. Cross-sectional associations were estimated via multivariable linear regression models. The overall GOHAI score was modeled as a three-level categorical variable (score 57–60: reference); the three subscores were modeled as continuous variables. Model 1 was adjusted for sex, age (continuous variable), marital status (married/cohabiting or living alone), education (up to high school, undergraduate or graduate degree), occupation (unemployed/student/homemaker/disabled, manual/blue collar, office work/administrative staff, professional/executive staff or retired), monthly household income (four-category variable), physical activity level (low, moderate, vigorous), smoking status (never, former or current smoker), alcohol use (g ethanol per day, continuous variable), total energy intake (kcal/day, continuous variable), and interval (in years) between sociodemographic and oral health data collection. Model 2 was additionally adjusted for BMI (continuous variable) and incident or prevalent cancer (any site), diabetes (self-report and/or anti-diabetic drug use), hypertension (self-report and/or antihypertensive drug use), and major cardiovascular disease (myocardial infarction, stroke, or acute coronary syndrome).

Because both oral health and dietary intake (including diet quality) likely vary by age [27,30], we performed tests for interaction between GOHAI score and age. All tests were two-sided and *p* < 0.05 was considered statistically significant. All analyses were conducted with SAS (version 9.4, SAS Institute, Inc., Cary, NC, USA).

## 3. Results

### 3.1. Sample Characteristics

**Figure 1:**
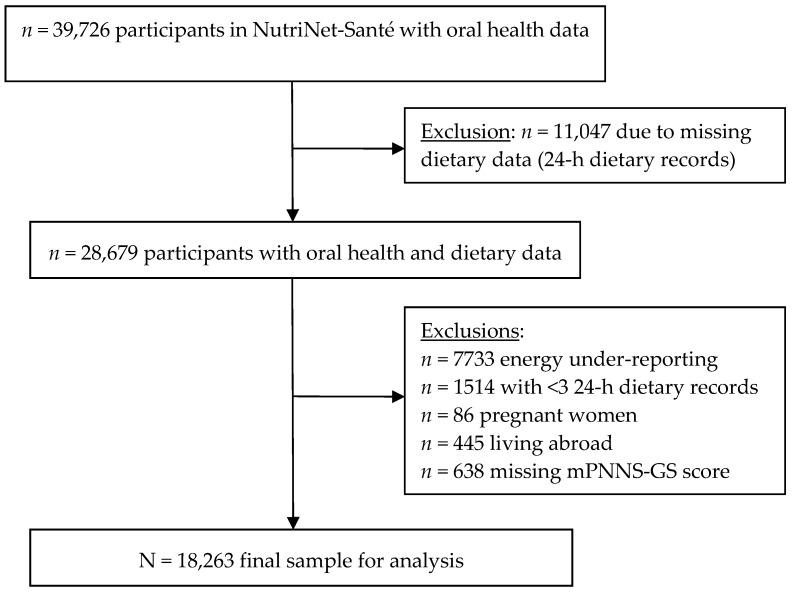
Participant selection flowchart.

Participant selection is summarized in Figure 1; the final sample included 18,263 individuals (4883 men; 13,380 women).

Compared with individuals excluded from the analysis, those who were included were more likely to be older and represented a slightly higher proportion of men; also, included participants had more favorable health behavior profiles (less likely to be current smokers, lower BMI, less alcohol use, slightly higher GOHAI scores). Mean age in the sample was 56.5 ± 13.8 years and mean overall GOHAI score was 54.5 ± 4.3. Mean mPNNS-GS score was 7.7 ± 1.6 and it was normally distributed in the full sample. Descriptive characteristics by the three-level GOHAI score are presented in Table 1.

We observed a significant inverse correlation between the overall GOHAI score and age, and a non-significant correlation between the overall GOHAI score and sex. Higher GOHAI scores were correlated with a higher educational level, higher monthly income, less current smoking, and better health profiles (lower mean BMI, less cancer, diabetes, or hypertension). There was a significant correlation between overall GOHAI score and mPNNS-GS (Table 2).

However, a non-significant correlation between overall GOHAI score and macronutrient intake (as percentage of total energy intake) was found. Older participants were somewhat more likely to adhere to dietary guidelines than were their younger counterparts (mPNNS-GS scores 8.0 ± 1.6 versus 7.6 ± 1.6, *p* < 0.0001; data not tabulated).

### 3.2. Association between Oral Health-Related QOL and Diet Quality

Owing to a statistically significant interaction between the overall GOHAI score and age (*p* < 0.01), we performed age-specific analyses (18–64 years versus 65+ years; Table 3).

Regarding the overall GOHAI score, modest, age-dependent associations between oral health and diet quality in partially and fully adjusted models were observed. In the younger age group, those scoring ≤ 50 on GOHAI were less likely to adhere to dietary guidelines than were participants scoring 57–60 points (Model 2: β = −0.18, 95% CI: −0.26, −0.09; *p* < 0.0001). In the older age group, those scoring 51–56 points on GOHAI were less likely to adhere to dietary guidelines than were participants scoring in the range 57–60 (Model 2: β = −0.23, 95% CI: −0.33, −0.13; *p* < 0.0001).

Next, the weak yet significant positive associations of mPNNS-GS with the physical function and the psychosocial function subscores did not differ by age in fully adjusted models. The association regarding discomfort/pain became non-significant following adjustment for health status covariates.

## 4. Discussion

This study provided some evidence of the association between oral health and diet quality in a large, heterogeneous sample of adults. The principal finding—in line with our hypothesis—was a significant albeit modest age-dependent association between oral health QOL and adherence to dietary guidelines. Among younger participants (ages 18–64 years), those scoring ≤50 on GOHAI (poor oral health with a detrimental impact on QOL) were less likely to adhere to dietary guidelines (as measured by mPNNS-GS) than were their peers scoring in the range 57–60 (good oral health). Among older participants (ages 65+ years), those scoring 51–56 points on GOHAI (average oral health with some negative impact on QOL) were less likely to adhere to dietary guidelines than were their peers scoring 57–60 points. It should be noted that extant research in the oral health-diet domain has dealt predominantly with the elderly. The findings could thus provide impetus to the study of QOL and other oral health aspects across age on the population level.

As expected and consistent with prior reports [19,20], younger individuals exhibited slightly higher GOHAI scores than did their older counterparts. Despite the high mean GOHAI scores in the two age groups studied, the values showed non-negligible variability, which was somewhat more pronounced among adults aged 65 years and over. Our findings in that age group suggest that with advancing age, even small unfavorable changes in oral health status (regarding physical and/or psychosocial function and/or discomfort/pain) could have a measurable negative impact on diet quality, independent of the role of general health status, socioeconomic status (financial resources, education, occupation), and other health/risk behaviors (smoking, alcohol use, physical activity). Diet quality was average, albeit somewhat higher among older than among younger participants, indicating that improvement across age and oral health status is warranted. Findings from four European countries had also shown that diet quality among the elderly was both relatively low on average yet heterogeneous across individuals [31]. Among community-dwelling U.S. elderly it was observed that at least 96% exceeded the guidelines (measured by HEI-2005) regarding saturated fat and sodium [13]. Our results are likewise congruent with those of a U.S. study with a nationally-representative sample of adults aged 60+ where having functional dentition (≥21 natural teeth) did not contribute substantially to higher HEI scores or nutrient intakes, yet irrespective of dentate status, diets of both sexes needed improvement [32].

The observed age-specific associations have clinical relevance in light of the ongoing worldwide demographic shift driven by rapid population aging [33]. The latter is one of the principal factors, along with unhealthy diet, physical inactivity, and substance use (tobacco, alcohol), contributing to the rise in the prevalence of chronic diseases [34]. The aging process is underscored by a host of physiological, functional, and health status changes all of which impact nutrition needs and dietary intake [35]. For example, the elderly have been shown to display a tendency to adapt their diets in response to functional difficulties, often leading to inadequate nutrient intakes [36].

The analysis did not reveal any significant differences across GOHAI categories regarding macronutrient intake quantity (percentage of total energy intake), whereas overall diet quality (mPNNS-GS) was significantly correlated with oral health (GOHAI). It is possible that differences in macronutrient intake were nuanced and only evident on a subcategory level. The modest associations observed in this analysis could be interpreted in the broader context of individual determinants of eating habits. A literature review recently reported that masticatory ability explained only part of the variance in food and nutrient intake among the elderly [37]. Likewise, in a sample of U.S. adults aged 37–81 years, masticatory performance was not a significant predictor of diet quality, measured by HEI [30]. Findings from the Canadian NuAge study with elderly individuals in relatively good heath indicated that diet quality (measured by the Canadian HEI) had not only oral health determinants (wearing dentures, chewing problems), but also various sociodemographic and lifestyle determinants [38]. Among German individuals aged ≥18 years, diet quality (assessed via an index constructed by the authors) was positively associated with age, education, income, engagement in sports, and food diversity [39]. Among Australian individuals aged ≥18 years, diet quality (assessed via HEI for Australians), was decreased among males, young adults, those who were obese, smokers, or were of low socioeconomic status [40].

Our findings were less pronounced regarding the three GOHAI dimensions compared with the overall score. We observed significant albeit very weak associations with the physical function and the psychosocial function subscores. In the older age group only, we also observed an association with the discomfort/pain subscore, which was driven by health status. Regarding value distribution, mean subscores in the sample were similar to those reported by a study among non-institutionalized elderly [41]. However, research linking any of the three subscores with nutrition or diet-related variables in non-clinical, non-institutionalized samples is lacking.

In the context of a complex, interdependent association among oral health, diet, and nutrition [42], a limitation of this study was its cross-sectional design, preventing inference of causality. Next, the GOHAI has discriminant potential regarding nutritional status [15], yet it cannot be used for dental disease diagnosis [19]. Diet quality was measured with the mPNNS-GS which accounts for food group rather than nutrient intake; in a given food group, nutrient composition might display notable variability [43]. Further, the sample included self-selected, likely health-conscious volunteers, which might explain the relatively high mean GOHAI score. The majority of the working French population has access to affordable dental care, even though no organized national strategies for oral health promotion exist [44]. Moreover, many volunteers were excluded from the analysis due to energy under-reporting or data completeness/quality issues. All of these aspects suggest the potential of selection bias and necessitate caution when generalizing the findings. Finally, the two age groups studied (18–64 years and 65+ years) were sociodemographically heterogeneous, possibly obscuring differences in oral health and diet. Nonetheless, important strengths of the study include its very large sample recruited from the general population, use of oral health and diet quality indices with established validity, and statistical control for many potential confounders.

Recent research has suggested that oral screening at midlife ought to be regarded as just as important as screening for hypertension and hypercholesterolemia, in view of premature mortality reduction on the population level [45]. Oral health promotion is likewise critical from an economic viewpoint, given that traditional curative dental care constitutes a heavy economic burden for many high-income countries [1]. The burden of oral and other chronic diseases might be decreased simultaneously by addressing common factors, such as maintaining oral hygiene, decreasing sugar intake, maintaining diet quality to prevent tooth decay, decreasing alcohol use to protect against oral cancer and periodontal disease, and smoking cessation [1]. Whereas the findings help advance knowledge in the fields of nutrition, oral health, QOL, and health behavior research, the observed age-specific associations were modest and merit confirmation in longitudinal research accounting for diet quality evolution, preferably using nationally representative samples.

## Figures and Tables

**Table 1:** Descriptive characteristics of study participants by overall GOHAI score (Etude NutriNet-Santé, N = 18,263).

	GOHAI Score						*p* ^1^
	57–60 (*n* = 7233)		51–56 (*n* = 7667)		≤50 (*n* = 3363)		
Sex							0.63
Men	1909	(26.4)	2058	(26.8)	916	(27.2)	
Women	5324	(73.6)	5609	(73.2)	2447	(72.8)	
Age, mean (SD)	54.3	(14.2)	56.4	(13.6)	59.8	(12.5)	<0.0001
GOHAI scores, mean (SD)							<0.0001
Overall score	58.4	(1.1)	53.9	(1.7)	47.3	(2.8)	
Physical function subscore	19.9	(0.3)	19.0	(1.3)	16.6	(1.7)	
Psychosocial function subscore	24.3	(0.8)	21.9	(1.6)	18.8	(2.0)	
Discomfort/pain subscore	14.2	(0.8)	12.9	(1.3)	11.2	(1.5)	
Marital status							0.0003
Married/cohabiting	5554	(76.8)	5708	(74.4)	2479	(73.7)	
Living alone	1679	(23.2)	1959	(25.6)	884	(26.3)	
Educational level							<0.0001
Up to high school	2012	(27.8)	2377	(31.0)	1385	(41.2)	
Undergraduate degree	2263	(31.3)	2403	(31.3)	1003	(29.8)	
Graduate degree	2958	(40.9)	2887	(37.7)	975	(29.0)	
Occupational status							<0.0001
Manual/blue collar	885	(12.2)	877	(11.4)	382	(11.4)	
Office work/administrative	1172	(16.2)	1140	(14.9)	386	(11.5)	
Professional/executive staff	1765	(24.4)	1627	(21.2)	488	(14.5)	
Retired	2788	(38.6)	3361	(43.8)	1790	(53.2)	
Household income (monthly)							<0.0001
<1200 €	553	(7.7)	578	(7.5)	297	(8.8)	
1200 €–1799 €	1253	(17.3)	1416	(18.5)	734	(21.8)	
1800 €–2699 €	1909	(26.4)	2040	(26.6)	901	(26.8)	
≥2700 €	2606	(36.0)	2666	(34.8)	979	(29.1)	
Missing/no response	912	(12.6)	967	(12.6)	452	(13.4)	
Physical activity ^2^							0.06
Low	2701	(37.3)	2829	(36.9)	1339	(39.8)	
Moderate	2965	(41.0)	3196	(41.7)	1332	(39.6)	
Vigorous	1567	(21.7)	1642	(21.4)	692	(20.6)	
Smoking status							<0.0001
Never	3976	(55.0)	3836	(50.0)	1513	(45.0)	
Former	2693	(37.2)	3149	(41.1)	1542	(45.8)	
Current	564	(7.8)	682	(8.9)	308	(9.2)	
Body Mass Index, kg/m^2^, mean (SD)	23.7	(4.1)	23.8	(4.2)	24.5	(4.6)	<0.0001
Diabetes ^3^	191	(2.6)	252	(3.3)	173	(5.2)	<0.0001
Cancer ^4^	656	(9.3)	808	(10.7)	394	(12.0)	0.0002
Hypertension ^5^	391	(5.4)	439	(5.7)	234	(7.0)	0.01
Major cardiovascular disease ^6^	107	(1.5)	114	(1.5)	79	(2.4)	0.0009
Interval between sociodemographic and oral health data collection, years, mean (SD)	3.7	(5.4)	3.7	(5.5)	3.7	(5.2)	0.96

Values refer to number (%) except when noted otherwise; GOHAI, General Oral Health Assessment Index. ^1^ *p*-values obtained from chi-squared tests and ANOVA, as appropriate. ^2^ Assessed with the International Physical Activity Questionnaire-Short Form; scoring followed established protocol. ^3^ Prevalent or incident diabetes type 1 or type 2 based on self-report and/or report of anti-diabetic drug use. ^4^ Prevalent or incident cancer (any site) based on data validated by a medical expert committee. ^5^ Prevalent or incident hypertension based on self-report and/or report of antihypertensive drug use. ^6^ Prevalent or incident major cardiovascular disease (myocardial infarction, stroke, acute coronary syndrome) based on data validated by a medical expert committee.

**Table 2:** Dietary intake and diet quality by overall GOHAI score (Etude NutriNet-Santé, N = 18,263).

	GOHAI Score						*p*
	57–60 (*n* = 7233)		51–56 (*n* = 7667)		≤50 (*n* = 3363)		
Total energy, kcal/d	1905.2	(494.3)	1912.0	(491.5)	1906.5	(494.3)	0.68
% energy from fat	40.5	(6.4)	40.3	(6.4)	40.2	(6.6)	0.15
% energy from protein	17.4	(3.5)	17.5	(3.4)	17.5	(3.5)	0.72
% energy from carbohydrates	42.1	(7.2)	42.2	(7.3)	42.3	(7.2)	0.67
Diet quality (mPNNS-GS)	7.8	(1.6)	7.7	(1.6)	7.7	(1.6)	0.02
Number of 24-h dietary records	3.9	(1.3)	3.8	(1.3)	3.9	(1.3)	0.91

**G**OHAI, General Oral Health Assessment Index; mPNNS-GS, modified Programme National Nutrition Santé—Guideline Score; values refer to mean (SD); *p*-values obtained from ANOVA.

**Table 3:** Age-specific linear regression models of the association between oral health-related quality of life and diet quality (Etude NutriNet-Santé, N = 18,263).

	18–64 Years			≥65 Years		
	β	95% CI	*p*	β	95% CI	*p*
Overall GOHAI score						
*Model 1*						
Score ≤ 50	−0.09	(−0.17, −0.00)	0.047	−0.07	(−0.16, 0.02)	0.11
Score 51–56	−0.03	(−0.09, 0.04)	0.432	−0.26	(−0.36, −0.16)	<0.0001
Score 57–60	(reference category)			(reference category)		
*Model 2*						
Score ≤ 50	−0.18	(−0.26, −0.09)	<0.0001	−0.08	(−0.16, 0.01)	0.08
Score 51–56	−0.05	(−0.11, 0.01)	0.077	−0.23	(−0.33, −0.13)	<0.0001
Score 57–60	(reference category)			(reference category)		
GOHAI physical function						
*Model 1*	0.01	(−0.01, 0.03)	0.169	0.05	(0.03, 0.07)	<0.0001
*Model 2*	0.03	(0.01, 0.04)	0.005	0.04	(0.02, 0.06)	0.0002
GOHAI psychosocial function						
*Model 1*	0.02	(0.00, 0.03)	0.016	0.04	(0.02, 0.05)	<0.0001
*Model 2*	0.03	(0.02, 0.04)	<0.0001	0.04	(0.02, 0.05)	<0.0001
GOHAI discomfort/pain						
*Model 1*	−0.00	(−0.02, 0.02)	0.718	0.03	(0.00, 0.05)	0.03
*Model 2*	0.01	(−0.01, 0.03)	0.324	0.02	(0.00, 0.04)	0.06

**G**OHAI, General Oral Health Assessment Index; diet quality modeled with mPNNS-GS, modified Programme National Nutrition Santé—Guideline Score. Model 1 adjusted for sex, age, marital status, educational level, occupational status, monthly household income, physical activity, smoking status, alcohol use, total energy intake, and interval between sociodemographic and oral health data collection; Model 2 additionally adjusted for BMI and incident or prevalent cancer, diabetes, hypertension, and major cardiovascular disease.
